# Intraoperative Fluorescein Staining of Cryopreserved Amniotic Membrane Grafts to Improve Visualization During and After Pterygium Surgery: A Novel Technique

**DOI:** 10.1097/ICO.0000000000000725

**Published:** 2016-01-08

**Authors:** Jenny Ha, J. Alberto Martinez, Michael Korchak, Sandra L. Cremers

**Affiliations:** * Department of Research, Visionary Eye Doctors, Rockville, MD;; † Department of Ophthalmology, Medstar Georgetown University Hospital, Washington, DC; and; ‡ Department of Ophthalmology, Medstar Washington Hospital Center, Washington, DC.

**Keywords:** amniotic membrane, fluorescein, pterygium

## Abstract

**Purpose:**

To describe a new method of enhancing the visualization of amniotic membrane grafts with fluorescein staining during pterygium surgery.

**Methods:**

Pterygium excision surgery using intraoperatively stained cryopreserved amniotic membranes was performed on 346 eyes. A sterile 0.6 mg sodium fluorescein strip was placed directly onto the amniotic membrane in the manufacturer's original packaging, and the stained allograft was then transplanted onto the planned site. Staining intensities, at 3, 5, and 10 minutes of dye immersion, were compared. Immediate postoperative pain rating (scale 0–10), visibility of the fluorescein-stained amniotic membrane graft, and conjunctival autograft and amniotic membrane graft elevation, dehiscence, retraction, or displacement were recorded. The recurrence rate of the study population was compared with that of a previous cohort of 121 patients who underwent pterygium excision with conjunctival autograft without stained amniotic membrane.

**Results:**

Direct contact of the fluorescein strip on the amniotic membrane at 3, 5, and 10 minutes showed no differences in subjective staining intensity. Fluorescein-stained amniotic membrane was easily detected on the ocular surface during and 24 hours after pterygium surgery. The average immediate postoperative pain rating was 0.8 ± 1.8. No intraoperative complications or postoperative amniotic membrane graft dehiscence, retraction, or displacement occurred. The recurrence rate using fluorescein-stained amniotic membrane (3 patients, 0.9%, mean follow-up time 31.8 ± 18.6 weeks) did not differ from that of the previous cohort without the stained amniotic membrane (2.5%; χ^2^(1) = 1.837, *P* = 0.183).

**Conclusions:**

Fluorescein strip staining of the amniotic membrane is a novel and safe intraoperative method to enhance visualization and handling of the graft during and after ocular surgeries.

## Abstract 2

Supplemental Digital Content is Available in the Text.

Pterygium is a common ocular surface disease characterized by cellular proliferation, neovascularization, and aggressive fibroblast proliferation.^1^–^3^ The indications for surgery include reduced vision due to encroachment on the visual axis, irregular astigmatism, chronic irritation, persistent inflammation, restricted ocular motility, and cosmetic concerns. Numerous surgical techniques have been described, including bare sclera excision, limbal and bulbar conjunctival autograft transplantation, and amniotic membrane transplantation, with or without the use of adjuncts, such as mitomycin C, radiation, and antiangiogenic agents.^4^

The human amniotic membrane graft is an allograft harvested from the innermost layer of the placenta, with antiinflammatory,^5^ antifibrotic, and antiangiogenic^6^ properties. It is approved by the Food and Drug Administration for wound covering and healing. Amniotic membrane grafts are commonly used in a spectrum of ocular surface disorders, such as corneal ulcers, superficial punctate keratitis, chemical and thermal burns of the cornea,^7^,^8^ persistent epithelial defects,^9^ symblepharon removal,^10^ fornix reconstruction,^10^ conjunctival replacement after conjunctival lesion removal,^11^ and pterygium excision surgery.^12^

According to our experience in pterygium surgery, the translucency of the amniotic membrane can limit a surgeon's visualization of the membrane and hinder its efficient handling (Fig. 1). We hypothesized that staining a cryopreserved amniotic membrane graft with fluorescein before transplantation would effectively aid visualization, due to its epithelial devitalization, and thus facilitate intraoperative manipulation. We describe a simple and efficient fluorescein-staining technique that facilitates visualization and manipulation of the amniotic membrane graft.

**FIGURE 1:**
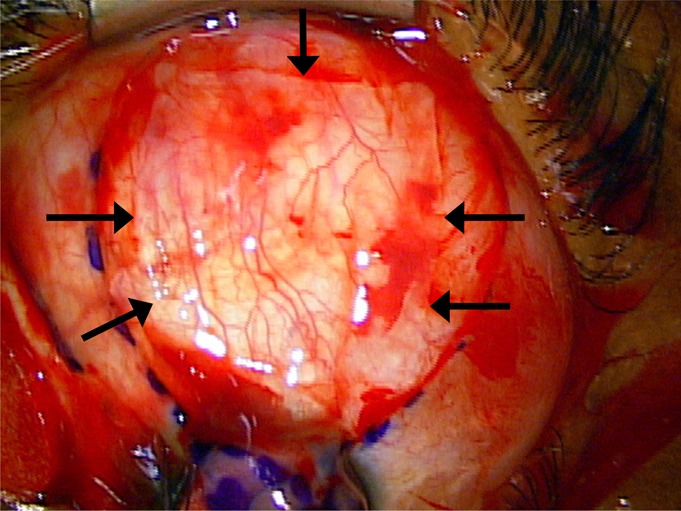
Unstained amniotic membrane removed from its original packaging and placed onto the surgical field. The arrows point to borders of the amniotic membrane; the anterior border is difficult to distinguish.

## MATERIALS AND METHODS

This retrospective study was approved by the Institutional Review Board of the Medstar Health Research Institute and Visionary Ophthalmology Ethics Committee. The surgical narratives and medical records of 346 pterygiectomy patients were reviewed. A surgical assistant placed a sterile 0.6 mg fluorescein sodium strip (FUL-GLO, Akorn Inc, Lake Forest, IL) in direct contact with the cryopreserved amniotic membrane (Amniograft, Bio-Tissue, Inc, Miami, FL) in the graft's original packaging, which contains a 2.5 mL solution of 1:1 glycerin:Dulbecco Modified Eagle Medium (equivalent to 0.024% fluorescein). In a subset of 4 cases, we compared the staining intensity of the stained allografts at 3, 5, and 10 minutes of dye immersion with a fluorescein strip; these results were also compared with 10 minutes of dye immersion with 6 drops of fluorescein sodium solution (Fluorescein Sodium 0.25% and Benoxinate Hydrochloride Ophthalmic Solution USP 0.4%; Bausch and Lomb, Rochester, NY). Simultaneously, the surgeon prepared the recipient site and obtained hemostasis by grabbing bleeding vessels with 0.12 mm forceps when necessary. No cautery was used.

**FIGURE 2:**
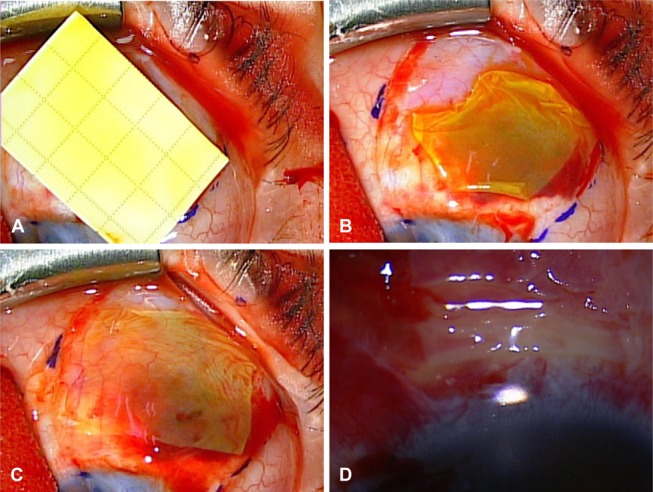
A, Stained amniotic membrane from its original packaging with the fluorescein strip onto the surgical field. B, Initial placement of the fluorescein-stained amniotic membrane graft before flattening and manipulation. C, Flattened fluorescein-stained amniotic membrane graft over a conjunctival defect. D, Amniotic membrane with visible staining 24 hours postoperatively.

Two experienced surgeons (J.A.M. and S.L.C.) performed pterygium surgery with the fluorescein-stained cryopreserved amniotic membrane transplanted onto different recipient sites after pterygium excision and extensive tenonectomy: over the superior bulbar conjunctiva autograft donor site for single pterygium or a nasal conjunctival defect for double-headed pterygia (J.A.M.) or over the rectus muscle underneath a conjunctival autograft (S.L.C.). The stained graft was placed directly onto the recipient site with fibrin sealant (TISSEEL; Baxter International Inc, Westlake Village, CA) (Fig. 2A). In a subset of 185 patients, pain ratings using a visual analog scale (0–10)^13^–^15^ were recorded. Postoperative steroid and antibiotic regimen was not altered with this new technique. The 24-hour postoperative visibility of the amniotic membrane graft was evaluated. Incidences of the conjunctival autograft and amniotic membrane graft elevation, dehiscence, retraction, and displacement were recorded.

Pterygium recurrence was defined as a new fibrovascular growth across the limbus originating from the conjunctival location of the previously excised pterygium. Statistical analyses were performed using SPSS Version 23 for Windows (SPSS Inc, Chicago, IL). The χ^2^ test was used to compare the recurrence rates between the current study population and those of a past cohort of 121 cases operated on by J.A.M. using unstained amniotic membrane grafts (June 2013–May 2014).

## RESULTS

This fluorescein staining technique has been performed on 346 pterygium excision cases since June 2014. There were no intraoperative complications. The average time of surgery was 19.3 ± 3.4 minutes. There was no subjective staining intensity difference between 3, 5, and 10 minutes of dye immersion with the fluorescein strip. Six drops of fluorescein sodium solution in the amniotic membrane packaging, 10 minutes before graft transplantation, resulted in subjectively less intensive staining than 3 minutes of fluorescein strip use.

Intraoperatively, the edges and folds of the fluorescent amniotic graft were highlighted and easily distinguished from the surrounding tissues (Fig. 2B). Accurate graft placement and thorough flattening with respect to the defect were easily achieved, eliminating the need for continuous manipulation to find the edges of the graft (Fig. 2C and see Video, Supplemental Digital Content 1, http://links.lww.com/ICO/A379). There was no detectable difference in the degree of staining between the basement membrane and stromal side of the amniotic membrane.

The average pain rating immediately after surgery was 0.8 ± 1.8 of 10. The amniotic membrane grafts were fluorescent 24 hours after staining with the fluorescein strip (Fig. 2D). There was no notable increase in the postoperative conjunctival autograft and amniotic membrane graft elevation, dehiscence, retraction, or displacement. No patients reported allergic reactions or unsatisfactory cosmetic appearance due to fluorescein-stained amniotic membranes. In addition, there was no increase in observed dellen formation or healing time. The recurrence rate after pterygium surgery with a conjunctival autograft and fluorescein-stained amniotic membrane (3/346 or 0.9%, mean follow-up time 31.8 ± 18.6 weeks) did not differ from that of pterygium surgery with an unstained amniotic membrane (3/121 or 2.5%; χ^2^(1) = 1.837, *P* = 0.183).

## DISCUSSION

This article describes the first report of using a fluorescein sodium strip to stain cryopreserved amniotic membranes intraoperatively. The efficient approach of using a fluorescein-stained amniotic membrane graft does not require graft rinsing before placement, aids manipulation of the amniotic membrane graft, and provides in vivo visualization during surgery and 24 hours after the procedure.

Previous in vitro human amniotic membrane fluorescein staining studies reported unsuccessful^16^ initial staining and dye dissipation at 24 hours.^17^ To assess which stain is ideal for facilitating intraoperative visualization and handling of human amniotic membranes, Hu et al^16^ and Kandavel and Chuck^17^ tested varying concentrations of indocyanine green, fluorescein sodium, lissamine green B, Rose Bengal, and trypan blue. Hu et al^16^ reported unsuccessful initial staining of freeze dried (IOP, Inc, Costa Mesa, CA) and cryopreserved (Bio-Tissue, Inc) amniotic membranes using 2 to 3 drops of fluorescein sodium solution (0.25%). Kandavel and Chuck^17^ immersed dry deepithelialized human amniotic membrane (OKTO Ophtho, Inc, Costa Mesa, CA) in 2 mL of dye for 30 seconds, rinsed the stained membrane with 15 mL saline solution, and reported staining loss after 120 minutes, 210 minutes, and 24 hours with fluorescein sodium 0.1%, 0.5%, and 1.0%, respectively. Neither study reported positive fluorescein staining at 24 hours.^16^,^17^ In both studies, fluorescein demonstrated the least staining intensity and duration, which may have been due to the use of prediluted fluorescein sodium solution, short duration of staining, routine rinsing of the stained membrane with saline starting 1 minute after contact with fluorescein, and routine exchanging of the saline storage media in 30-minute intervals. The direct contact of the fluorescein strip in the manufacturer's original packaging with the amniotic membrane, may have facilitated the greater staining intensity observed in this study.

Since June 2014, we have performed this new technique on 346 cases of pterygium removal with amniotic membrane transplantation. Although Kandavel and Chuck stated that fluorescein may be a good intraoperative dye because of its dissipation in less than 24 hours, a stained amniotic membrane graft is useful in monitoring its placement 24 hours after the procedure. The postoperative visibility of fluorescein-stained amniotic membrane grafts is likely due to the localized and prolonged fluorescein soaking during graft preparation and the lack of irrigation after graft application. The dye dissipates during the early postoperative period, between 24 hours and 7 days, likely from the administration of ophthalmic medications and natural tear production.

Our recurrence rate of 0.9% is among the lowest recently reported rates (0.1–15.4%).^18^–^25^ Adverse intraoperative and postoperative reactions to fluorescein have not been observed. In addition, there have been no noted patient complaints regarding the stained amniotic membranes postoperatively. Future research will determine whether postoperative parameters, such as subconjunctival hemorrhaging, scar formation, and final cosmetic outcomes, are equivalent between fluorescein-stained amniotic membranes and non–fluorescein-stained amniotic membranes.

In conclusion, our novel technique of staining cryopreserved amniotic membrane grafts with a fluorescein strip safely enhances visualization and facilitates manipulation of the amniotic membrane intraoperatively. The stained amniotic membrane is visible during the first 24 hours after surgery with no apparent adverse consequences. Fluorescein-stained amniotic membrane may prove useful in other ocular procedures.

## Supplementary Material

SUPPLEMENTARY MATERIAL
